# Generalisation of New Sequence Knowledge Depends on Response Modality

**DOI:** 10.1371/journal.pone.0053990

**Published:** 2013-02-05

**Authors:** Clive R. Rosenthal, Tammy W. C. Ng, Christopher Kennard

**Affiliations:** 1 Nuffield Department of Clinical Neurosciences, University of Oxford, Oxford, United Kingdom; 2 Department of Anaesthetics, University College London Hospitals NHS Foundation Trust, University College London, London, United Kingdom; Harvard Medical School, United States of America

## Abstract

New visuomotor skills can guide behaviour in novel situations. Prior studies indicate that learning a visuospatial sequence via responses based on manual key presses leads to effector- and response-independent knowledge. Little is known, however, about the extent to which new sequence knowledge can generalise, and, thereby guide behaviour, outside of the manual response modality. Here, we examined whether learning a visuospatial sequence either via manual (key presses, without eye movements), oculomotor (obligatory eye movements), or perceptual (covert reorienting of visuospatial attention) responses supported generalisation to direct and indirect tests administered either in the same (baseline conditions) or a novel response modality (transfer conditions) with respect to initial study. Direct tests measured the use of conscious knowledge about the studied sequence, whereas the indirect tests did not ostensibly draw on the study phase and measured response priming. Oculomotor learning supported the use of conscious knowledge on the manual direct tests, whereas manual learning supported generalisation to the oculomotor direct tests but did not support the conscious use of knowledge. Sequence knowledge acquired via perceptual responses did not generalise onto any of the manual tests. Manual, oculomotor, and perceptual sequence learning all supported generalisation in the baseline conditions. Notably, the manual baseline condition and the manual to oculomotor transfer condition differed in the magnitude of general skill acquired during the study phase; however, general skill did not predict performance on the post-study tests. The results demonstrated that generalisation was only affected by the responses used to initially code the visuospatial sequence when new knowledge was applied to a novel response modality. We interpret these results in terms of response-effect distinctiveness, the availability of integrated effector- and motor-plan based information, and discuss their implications for neurocognitive accounts of sequence learning.

## Introduction

Humans and experimental animals exhibit a prodigious capacity to learn complex regularities in the environment via visuomotor responses [1]. Once acquired, knowledge about these regularities can guide behaviour in novel situations [2], [3]. Behavioural tasks such as the serial reaction time task (SRT task) have been adopted in the laboratory to study the learning of regularities based on a repeating visuospatial sequence [4], [5]. Learning on the SRT task typically involves manual key presses directed to four fixed locations that are guided by a sequence of visual targets presented at four corresponding locations on a computer screen [4]–[7]. Over time, the reduction in the reaction time (RT) associated with the manual key presses is attributed to the emergence non-specific visuomotor learning (i.e., an improvement in general visually guided motor response execution), whereas sensitivity to predictable features of the visuospatial sequence – revealing sequence-specific knowledge mediated by rule-based learning – is inferred if there is an increase in RT, when a non-predictable sequence is presented. Sequence-specific knowledge has been shown to transfer readily between different (manual) motor outputs on the same hand, between different hands, and between finger and limb movements [8]–[14]. Therefore, the products of manual sequence learning can be independent of the initial effector and response used to code the visuospatial sequence [8], [15]–[26]; that is, the new knowledge can be specified in visuospatial (allocentric) coordinates. Importantly, however, this conclusion is based on studies that involved re-mappings of a manual effector between study and transfer. By comparison, little is known about the extent to which new sequence knowledge can generalise and thereby guide responses outside of the manual response modality.

Recent studies have demonstrated that visuospatial sequences can also be learned when the responses are confined to observation (perceptual sequence learning via sequential shifts of covert visuospatial attention) rather than action [27], [28]. However, it is currently unknown whether new knowledge acquired via perceptual sequence learning supports transfer onto a novel response modality. Visuospatial sequences can also be learned via eye movement responses, but the resultant knowledge is detectable only on tests that have informational and task demands which overlap closely with the original conditions of learning [6], [29]–[31]. Notably, prior studies have also failed to find evidence of transfer onto tests that require a novel mode of responding, with respect to initial study. In particular, in a study by Marcus et al. [29], knowledge acquired via combination of free eye movements and covert reorienting of visuospatial attention directed to a visuospatial sequence was detected in a change in the frequency of anticipatory eye movements, but learning did not support above baseline knowledge on a post-study manual SRT task, a free-generation task, or on a task that required participants to (verbally) predict upcoming locations. Relatedly, Albouy et al. [6] reported that learning via obligatory eye movements led to a non-specific reduction in saccade latency, but an increase in saccade latency on the presentation of a non-predictable (vs. predictable) sequence. After training, participants were unable, however, to self-generate eye movements or manual key presses guided by knowledge of the studied sequence. Finally, Kinder et al. [30] examined learning on an oculomotor SRT task where stimulus presentation was contingent on making obligatory saccades to four fixed locations. Although training led to sequence-specific learning, newly acquired knowledge was not reported when assessed on a post-study paper and pencil “old”/“new” recognition memory test.

**Figure 1:**
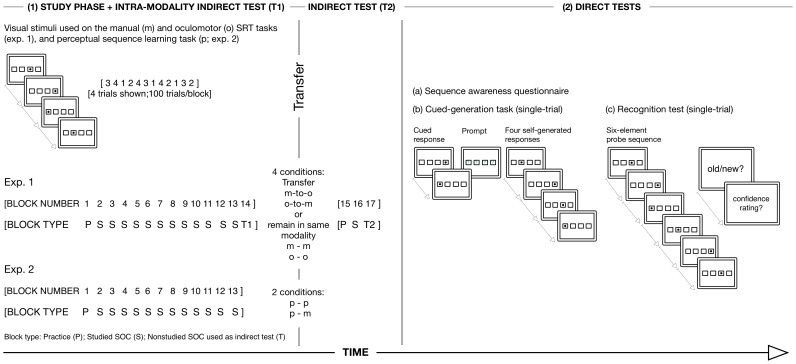
Experimental setup and design in experiments 1 and 2. (1) Study phase and indirect tests of sequence knowledge. Schematic shows the structure of the baseline (exp. 1: m-m, o-o; exp. 2: p-p) and transfer conditions (experiment 1: m-o, o-m; experiment 2: p-m). Responses to each visual target required a manual key press (exp. 1: manual SRT task, performed without eye movements), an obligatory eye movement (exp. 1: oculomotor SRT task, involving saccade-contingent stimulus offset), or a covert shift of visuospatial attention (exp. 2: perceptual sequence learning task). Indirect tests of sequence-specific knowledge (a block of trials based on a nonstudied second-order conditional sequence) were administered on blocks 14 (vs. 13 [target sequence block]) (T1, intra-response modality) and 17 (vs. 16 [target sequence block]) (T2, inter-response modality in m-o and o-m transfer conditions). P denotes a pseudorandom practice block of trials (blocks 1 and 15); S denotes a target sequence block; and, T denotes the indirect tests. (2) Post-study direct and indirect tests in the baseline and transfer conditions: (a) sequence awareness questionnaire; (b) schematic of single-trial event sequence used on manual, oculomotor, and perceptual versions of inclusion-exclusion cued-generation task. Each trial was comprised of two cues and four self-generated responses. Red (exclusion test) or green (inclusion test) question marks appeared (block-wise) after the two cues as the prompt to self-generate four manual (m-m, o-m, p-m), oculomotor (o-o, m-o) or vocal (p-p) responses that were either different from (exclusion test) or the same as (inclusion test) the studied sequence; and (c) schematic of the manual (m-m, o-m, p-m), oculomotor (o-o, m-o), and perceptual (p-p) versions of the “old”/“new” recognition memory test (12 studied/“old”; 12 nonstudied/“new”). Participants determined whether each six-element sequence was “old” or “new”, and then rated their confidence on a 6-point scale. In summary, participants in the baseline conditions continued to respond using versions of the tests that were implemented in the same response modality as the study phase and indirect tests (T1, T2). Participants in the transfer conditions continued to respond using versions of the tests that were implemented in a novel response modality with respect to the study phase.

Here, we examined whether new sequence knowledge acquired via manual key presses without eye movements (manual SRT task), obligatory eye movements (oculomotor SRT task), or covert reorienting of visuospatial attention (perceptual sequence learning task) could generalise and thereby guide responses on post-study tests administered either in the same (baseline conditions) or a novel response modality (transfer conditions) with respect to initial study (Figure 1). The visuospatial sequence was based on a deterministic second-order conditional rule that was specified at four fixed screen locations. At the lowest structural level, the ability to predict an upcoming location was dependent on learning two preceding target locations [32]. Hence, learning the visuospatial sequence per se, as opposed to non-specific (general visuo-) motor or response learning, was dependent on developing sensitivity to the higher-order (non-adjacent) associations specified at the level of three or more consecutive locations, rather than on the frequencies of individual locations or first-order (pairwise) locations.

New sequence knowledge was assessed using both indirect and direct tests. The indirect tests were based on an assessment of the relative differences in response latencies (manual RT, saccade latency) between studied and nonstudied sequences (i.e., predictable vs. non-predictable), and did not ostensibly draw on the study phase (Figure 1). By comparison, the direct tests referred to the study phase, and involved informational and task demands that were markedly different to those engaged at study. The first was a cued-generation task [28], which required participants to self-generate responses based on the studied sequence, under the two instructional conditions of the process-dissociation procedure [28], [33]. We used the cued-generation task to measure the ability of participants to exert conscious control over newly acquired sequence knowledge in a novel response context (the baseline conditions) and in a novel response modality (the transfer conditions). The second direct test was a forced-choice “old”/“new” recognition memory test [34]. Participants were presented with six-element sequences, which were based on the studied (old) sequence and on a nonstudied (new) sequence. Sensitivity to the available conscious knowledge was assessed by prompting participants to respond on a six-point confidence scale after each old/new discrimination [34]. The accuracy and response latency associated the six-element sequences provided concurrent direct and indirect measures, respectively [35]; the latency based analysis enabled us to determine the number of elements (i.e., the amount of environmental context) needed to induce response priming on the recognition test [36]. By using these multiple post-study tests, we were able to maximise the sensitivity to the available knowledge [6], [28], [37], [38], and, importantly, the assessment of conjoint performance across these tests provided an objective measure of the flexibility with which new knowledge could be deployed in the same (manual, oculomotor, and perceptual baseline conditions) or a novel (manual to oculomotor, oculomotor to manual, and perceptual to manual transfer conditions) response modality with respect to initial study.

We have previously shown that manual and perceptual sequence learning lead to knowledge that can be used on intra-response modality versions of the cued-generation task and recognition memory test [28]. Therefore, we predicted that we would replicate these results in the manual and perceptual baseline conditions, and predicted that learning on the oculomotor SRT task would support a comparable level of representational flexibility on the oculomotor post-study tests, because many common principles of function underscore manual and oculomotor modalities [39]. By contrast, in the transfer conditions, several factors were hypothesised to affect the use of newly acquired knowledge on the post-study tests [9], [40]. First, extended rehearsal on the manual SRT task can lead to effector-dependent knowledge, whereby the knowledge is specified in motor/response specific coordinates [16], [41], [42]. In agreement with these behavioural data, evidence from functional neuroimaging suggests that effector-dependent learning is, in fact, slower than effector-independent learning [43]. Therefore, even though our study protocol was equated across conditions in terms salient learning parameters, such as the response-to-stimulus interval and amount of exposure to the target visuospatial sequence, transfer in the manual to oculomotor and oculomotor to manual transfer conditions was not predicted to be equivalent. We reasoned that the lower relative demands on response selection associated with the oculomotor SRT task would reduce the rate of learning compared to the manual SRT task [44], [45]. Accordingly, oculomotor learning was predicted to be less vulnerable to the emergence of effector-dependent knowledge. Second, even though perceptual sequence learning can lead to knowledge that can be expressed on perceptual direct and indirect tests [28], we predicted nominal transfer onto the manual direct and indirect tests, because motor responses directed to a set of locations appear to be essential for visuomotor learning [26], [46], [47].

## Methods

### Participants

In experiment 1, sixty-four participants (M for age  = 20.8 [18–31 years]; 45 female) were assigned to one of four between-subjects conditions (n = 16/condition), whereas in experiment 2, thirty-two participants (M for age: 21.6 (range  = 18–25) years; 8 females) were assigned to one of two between-subject conditions (n = 16/condition). A total of 85 participants were recruited in experiment 1 (M = 21.4): data from 9 participants (4 female, 5 male) were unusable due to technical errors and data from 12 participants (2 female, 10 male) were unusable because a loss of eye tracking integrity led to early termination of the study phase. All participants had normal or corrected-to-normal visual acuity, and received a payment of £15. None of the participants had previous experience of the SRT task or sequence learning tasks.

### Ethics statement

Local research ethics committee (West London 1 Research Ethics Committee: 04/Q0406/147) approval was granted for the experimental procedures. All participants provided written informed consent for the collection of data and subsequent analysis.

### Visual stimuli

**Figure 2:**
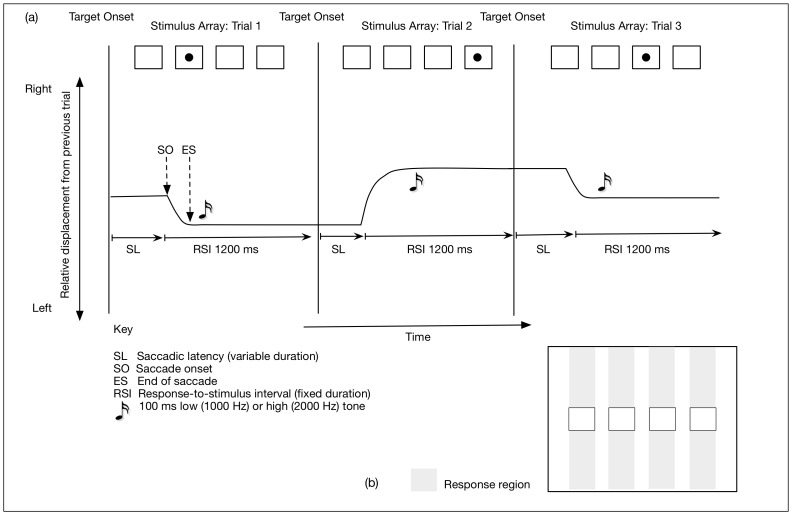
Schematic showing three trials of the oculomotor SRT task. (a) Participants were instructed to respond by directing their gaze to the location of each target stimulus. Saccade onset was detected in real time and automatically using a velocity criterion (the first two consecutive data points that corresponded to an increase in instantaneous velocity above 30° per second). A target stimulus remained on-screen for 100 ms after a response was detected, or, if no response was detected, the target was extinguished after 5000 ms had elapsed. If an incorrect response was detected, a 2000 Hz (100 ms) tone was played 50 ms after the end of the saccade (ES). (b) Saccade endpoints of the first saccadic response that landed within the full-screen vertical area subtended by the horizontal width of one of the four boxes containing the target were categorised as a correct response. Saccade endpoints that occurred outside one of the four designated response regions were categorised as a location error, and, as with a correct response, also terminated a trial.

Visual stimuli were presented on a 21-inch Sony CRT monitor – configured to a refresh rate of 100 Hz and screen resolution of 1024×768 pixels – controlled by a Dell PC running implementations of the experimental tasks written in C++. Target stimuli (circle 0.5 cm in diameter) appeared at the centre of one of four possible screen locations defined by squares outlined in black and aligned along the horizontal meridian on a white background, at a viewing distance of 75 cm (Figure 1). Eccentricities and dimensions of the four squares were the same on manual and oculomotor SRT tasks, perceptual sequence learning task, and all versions of the cued-generation task and recognition memory test (Figure 1). A 1200 ms response-to-stimulus interval (RSI; the delay between a participant pressing a button in response to a stimulus or detection of a saccade to the stimulus and the onset of the next trial) was used on the oculomotor and manual SRT tasks and recognition tests (Figure 1 and Figure 2).

### Visuospatial sequence

Each block of trials during the study phase was comprised of 100 trials (Figure 1). The first four trials were buffer trials and the next 96 trials consisted of eight repetitions of one of two 12-element second-order conditional sequences, which were generated using a deterministic second-order conditional (SOC) rule that, at the lowest structural level, ensured a position could be predicted by two previous locations [32] (SOC1: 3 4 2 3 1 2 1 4 3 2 4 1; SOC2: 3 4 1 2 4 3 1 4 2 1 3 2; screen locations are referred to as 1–4, read left to right). The sequences were equated along dimensions of location frequency, first-order transition frequency, reversal frequency, rate of full coverage, and contiguous repetitions of a location were excluded (the sequences are identical to those used by Destrebecqz and Cleeremans [37]). Half of the participants studied SOC1 and the other half were studied SOC2.

### Design

Both experiments were conducted in two main phases (Figure 1): (1) the study phase, which was comprised of 12 blocks, and involved a total of 96 repetitions of the 12-element second-order conditional sequence. Participants were trained on either a manual SRT task (exp. 1), an oculomotor SRT task (exp. 1), or a perceptual sequence learning task (exp. 2); and (2) the test phase. Sequence-specific knowledge in the test phase was examined using indirect tests that measured response priming associated with the studied sequence (indirect tests T1, T2 [exp. 1]; recognition priming [exps. 1 and 2]), and direct tests that measured self-generated responses informed by the studied sequence (cued-generation task) and recognition memory for the studied sequence. Response modality was manipulated as a between-subjects variable in both experiments (n = 16/condition). The direct and indirect tests were administered either in the same response modality (baseline m-m, o-o, and p-p conditions – the underlined letter indicates the test phase response modality) or in a novel response modality (transfer m-o, o-m, and p-m conditions) as the study phase.

#### (1) Study phase and indirect tests

In experiments 1 and 2, the target 12-element second-order conditional sequence was presented between blocks 2 to 13 (inclusive) (i.e., 12 blocks of study). Block 1 was a practice block and was comprised of a pseudorandom sequence of trials – contiguous repetitions of a single location were not presented and the four stimulus locations were balanced for frequency of occurrence. In experiment 1, the order of the stimuli on blocks 14 (Test 1 [T1]) and 17 (Test 2 [T2]) was determined by the non-predictable SOC. The non-predictable SOC on block 14 (T1) served as an intra-modality indirect test of sequence-specific knowledge in the baseline and transfer conditions, and block 17 served as an indirect test of the transfer sequence-specific knowledge in the m-o and o-m transfer conditions (T2). In the m-m and o-o baseline conditions, block 17 provided an assessment of whether sequence-specific knowledge could be detected on the second indirect test, when there was no change in response modality. Block 15 served as a practice block in the transfer conditions for responding in the new modality, and was also included in the baseline conditions.

#### (2) Direct tests

(a) A sequence awareness questionnaire was administered immediately after the two indirect tests and required the participant to selected one of five propositions. (b) The cued-generation task followed the awareness questionnaire and was comprised of an inclusion test and an exclusion test [28] – test order was counterbalanced across participants. The nonconscious use of knowledge was inferred if equivalent proportions of the target sequence were generated across inclusion (I) and exclusion (E) tests (I = E, or E>Baseline [B]) – intrusion errors on the exclusion test are assumed to reflect an inability to exert conscious control to withhold the new sequence knowledge from the responses. By contrast, if performance on the inclusion test exceeded the exclusion test (I>E), sequence knowledge was assumed to be conscious if performance exceeded baseline only on the inclusion test. Each cued-generation test consisted of 12 trials. Stimulus materials for each trial were generated by starting at each serial position of the studied 12-element SOC sequence for two consecutive positions. (c) The recognition memory test followed the cued-generation task and was comprised of 24 six-element sequences. Twelve sequences (starting from each ordinal position of the 12-element SOC sequence for six consecutive locations) were generated from SOC1 and 12 were generated from SOC2. A six-point scale was used to obtain a confidence rating for each six-element sequence [34].

### Procedure

All participants were tested individually and the experiments were performed in a dark visual Ganzfeld. Written instructions were presented on the monitor and were supplemented, where appropriate, with explanation provided by the experimenter. Head movements were minimised using a chin rest. In addition to familiarising participants with the SRT tasks and perceptual sequence learning task, the initial pseudorandom block of 100 practice trials (block 1) also encouraged incidental learning of the sequence (Figure 1).

#### (1) Study phase and indirect tests

Each trial of the manual SRT task, oculomotor SRT task, and perceptual sequence learning task was comprised of a single target stimulus presented at one of four possible locations (Figure 1).

#### Experiment 1 (manual SRT task and oculomotor SRT task)

In the manual SRT task, participants were instructed to press one of four buttons in response to the onset of each target (RB Series Response Pad, Model RB-730, Cedrus Corporation, California, USA), while maintaining central fixation; that is, participants were instructed to avoid making eye movements and rely on peripheral vision alone to detect each target. Responses in the manual SRT task were made to locations 1 and 2 with the middle and index fingers of the left hand, respectively, and to locations 3 and 4 with the index and middle fingers of the right hand, respectively. In the oculomotor SRT task, participants were instructed to respond to targets by directing their gaze to each target and maintain fixation until the onset of the next target (Figure 2). The target stimulus was extinguished on detection of a response and the next trial started, or, if no response was made by the participant, the target was extinguished after 5000 ms. Each target remained on the monitor for 100 ms after the saccade endpoint was detected (dwell time) on the oculomotor SRT task and indirect tests (T1; T2), or, 100 ms after a manual response was recorded in the manual SRT task and indirect tests (T1; T2) – the 100 ms dwell time was incorporated into the 1200 ms RSI (Figure 2). Participants were instructed to respond to the location of each target as quickly and as accurately as possible. Mismatch errors and location errors were immediately followed by a 2000 Hz tone (100 ms). Participants were informed about the change in response mode after block 14 of the study phase, and were instructed that block 15 was an opportunity to practice responding in a new response modality.

#### Experiment 2 (perceptual sequence learning task and large diameter target counting task [LDT counting task])

Participants were instructed to maintain central fixation and perform the LDT counting task concurrent to the perceptual sequence learning task. The LDT counting task encouraged attention to the sequence of visual targets. Two sizes of target-stimuli – standard and large diameter targets (LDTs) – were presented during the study phase: standard targets were circles 0.5 cm in diameter, whereas LDTs were 0.8 cm in diameter (1000 ms, 200 ms ISI). The order of the LDTs was random within a block and number per block was set at a proportion (18 and 36% of trials) that ensure performance was at ceiling [28]. Participants were required to maintain a cumulative blockwise count of LDTs and report the value at the end of each block. Block-by-block on-screen feedback on the accuracy of the value was provided. Participants entering a count within 5% accuracy were informed that their count was accurate and were asked to continue with their good performance, whereas participants responding with a count of 5% error or greater were shown their percentage underestimation or overestimation, and were instructed to try harder in the forthcoming block of trials. Visual targets were presented for 1000 ms and were separated by a 200 ms ISI.

#### (2) Direct tests

All participants were asked to select a proposition that best described their knowledge of the study phase, before moving onto the cued-generation task. Five propositions were presented: 1 =  “The sequence of stimuli was random”; 2 =  “Some positions occurred more often than others”; 3 =  “The movement was often predictable”; 4 =  “The same sequence of movement would often appear”; and 5 =  “The same sequence of movements occurred throughout the experiment” [48].

#### Cued-generation tasks (manual, oculomotor, and perceptual versions)

Each trial of the cued-generation task began with the presentation of two consecutive target stimuli (Figure 1). Then, depending on the block-wise manipulation of test instructions, either a green (inclusion test) or a red (exclusion test) question mark appeared in each of the four boxes, as a prompt to generate four responses that followed on from the two cues. In the inclusion cued-generation test, participants were instructed to generate responses that corresponded as closely as possible to the next four positions of the sequence presented during the study session, even if they felt they could not remember particular parts of the sequence that followed the cues. In the exclusion test, participants were instructed to generate follow-on sequences that were novel, and, therefore, avoided the sequence seen during the study phase. Hence participants were required to inhibit ‘‘prepotent’’ responses in order to generate a new (with respect to study) follow-on sequence. Participants were also asked to avoid generating follow-on sequences that were comprised of repetitions or natural sequences (e.g., 4-4-2-1 or 1-2-3-4). Each location was entered online by the experimenter and a circle appeared in the corresponding box.

In the manual cued-generation task (m-m, o-m, and p-m conditions), key presses were required to record responses to the two cues on each trial and indicate the locations of the self-generated follow-on sequence (confirmed by four black circles in the corresponding boxes). In the oculomotor cued-generation task (m-o, o-o conditions), participants were instructed to direct their gaze to the first two cues of each trial and maintain fixation until the target extinguished (extinction occurred only on detection of correct gaze direction). Self-generated responses were then required and involved participants directing their gaze to a box location and maintaining fixation until a black circle appeared in the corresponding box. In the perceptual cued-generation task (p-p condition), participants were instructed to direct their attention to the locations of the first two cues without moving their eyes and then provide four verbal responses related to the next four follow-on locations. Visual feedback for the follow-on locations appeared in the same way as on the manual and oculomotor cued-generation tasks.

After completion of each cued-generation test, participants were asked to indicate their confidence – on a scale of 0–100 – in being able to perform in accordance with the test instructions. The test level assessments of how confident participants were on the inclusion and exclusion tests were included to determine whether meta-knowledge about the studied sequence was necessary for accurate performance.

#### Recognition memory tests (manual, oculomotor and perceptual versions)

Manual and oculomotor versions required that the participants respond as quickly and as accurately as possible to each six-element probe sequence, using either key presses or obligatory eye movements, respectively. On the perceptual version, participants were asked to direct their attention to each six-element sequence, without making any eye movements. On all versions, participants then indicated whether each six-element sequence was “old” or “new”, followed by a response on a six-point scale to indicate the level of confidence associated with each old/new response [34]: 1 =  “I'm certain that this fragment was part of the training sequence”, 2 =  “I'm fairly certain that this fragment was part of the training sequence”, 3 =  “I believe that this fragment was part of the training sequence”, 4 =  “I believe that this fragment was not part of the training sequence”, 5 =  “I'm fairly certain that this fragment was not part of the training sequence”, and 6 =  “I'm certain that this fragment was not part of the training sequence”.

### Eye movement recording and monitoring

Saccade latency, accuracy, and central fixation were recorded using an Eyelink II infrared eye tracker (SR Research Ltd. Mississauga, Ontario, Canada; spatial resolution: <0.5°), which was controlled by an IBM-compatible PC. Eye dominance was determined for each participant using the near-far alignment test (experiment 1, right dominant: 47; experiment 2, right dominant: 22) [49]. Viewing was binocular. Monocular, pupil-only eye tracking of the dominant eye was conducted at a sampling rate of 250 Hz. Calibration and validation of eye position were performed at the beginning of each block of the manual and oculomotor SRT tasks, perceptual sequence learning task, and the beginning of each direct test.

#### Saccade latency

Saccade onset was detected automatically and in real time according to a velocity criterion defined as the first of two consecutive data points that corresponded to an increase in instantaneous velocity above 30° per second. Saccade latency and response accuracy of the first saccade (post-target onset) that met this criterion were used as the primary dependent measures on the oculomotor SRT task and oculomotor recognition test. By way of equivalence, RT on the manual SRT task was measured from the onset of the target-stimulus to the depression of a key on the response pad.

#### Accuracy

Saccade endpoints of the first saccade that occurred within the full-screen vertical area subtended by the horizontal width of one of the four squares that marked the target locations were categorised as one of two responses: (1) a correct response – the saccade endpoint and location of the target stimulus occurred within the same response region; or, (2) a mismatch error – the saccade endpoint occurred within a response region that did not contain the target stimulus (Figure 2). Saccade endpoints that did not fall within one of the four designated response regions were categorised as a location error – these errors were largely due to a loss of calibration that led to difficulty with maintaining sufficiently accurate eye tracking. Detection of all three categories of response terminated a trial.

#### Central fixation during the manual and perceptual behavioural tasks

Eye movements were monitored online by the experimenter to ensure that the participants did not deviate from central fixation. If the experimenter observed deviation, feedback was provided to restore central fixation.

#### Offline eye movement analyses of central fixation

In the m-m, m-o, o-m, p-p, and p-m conditions, eye movement fixation data were analysed off-line using a proprietary application developed within MATLAB® 6.5.1 (The Mathworks, Inc., Natwick, MA). Saccades with latency of 12 ms or less were excluded from a trial sample, as were saccades that began and ended within a central region (1°).

## Results

In the first two subsections below, we summarise the main results from the transfer conditions because these data speak directly to the capacity of sequence-specific knowledge acquired during manual, oculomotor, and perceptual sequence learning to generalise and guide responses in a new response modality. After these summaries, we provide a complete account of the results obtained on the individual indirect and direct tests administered in the baseline and transfer conditions. In these later sections, for example, we report that performance in the manual, oculomotor and perceptual baseline conditions revealed significant sequence-specific knowledge on the respective versions of the cued-generation tasks and recognition memory tests.

### Experiment 1 summary: significant transfer of sequence-specific knowledge after manual and oculomotor sequence learning (m-o and o-m conditions)

**Figure 3:**
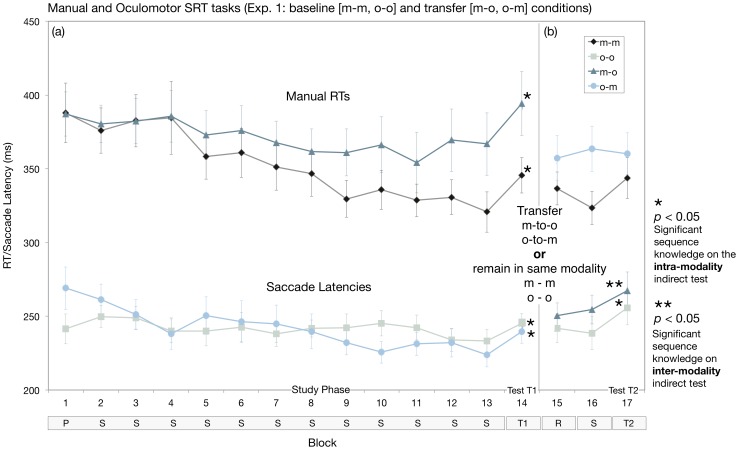
Mean reaction times and saccade latencies across manual and oculomotor SRT tasks, respectively, and two indirect tests of sequence-specific knowledge (T1 [intra-response modality]; T2 [inter-response modality in o-m and m-o transfer conditions]). (a) Significant sequence knowledge was evident in the baseline (m-m, o-o) and transfer (m-o, o-m) conditions on the first intra-modality indirect test (T1 [non-studied sequence], blocks 14 vs. 13). The magnitude of general skill acquired across the entire study phase on the manual SRT task was significantly different between the m-m and m-o conditions; however, this difference did not lead to a significant between-condition difference in the magnitude of sequence-specific knowledge (T1, T2), and the variability in general skill did not predict performance on the post-study tests administered either in the same or a different modality as initial study. (b) The second indirect test (T2) revealed evidence of sequence-specific knowledge in the m-m and o-o baseline conditions and significant transfer in the m-o, but not o-m, condition. P denotes a pseudorandom practice block of trials (blocks 1 and 15); S denotes the target sequence block; and T denotes the indirect tests, which were based on the same structural properties as the target sequence, but had a different ordinal sequence of locations. Error bars correspond the S.E.M.'s.

**Figure 4:**
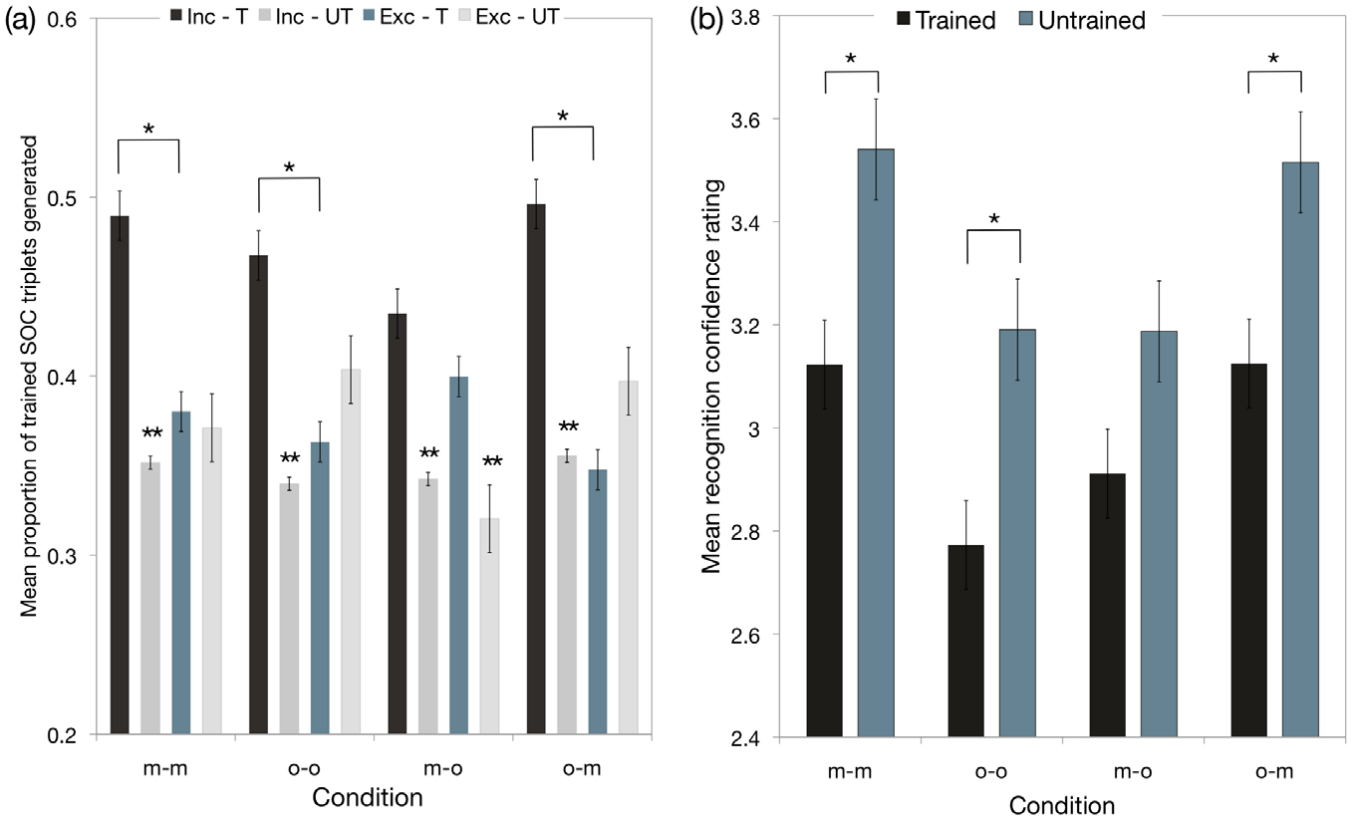
Performance on the cued-generation tasks and recognition memory tests administered in experiment 1. (a) Mean proportions of studied and nonstudied (baseline) second-order conditional triplets generated for m-m and o-o baseline and m-o and o-m transfer conditions, under inclusion and exclusion test instructions. Both baseline conditions revealed evidence of conscious control over self-generated responses after manual and oculomotor sequence learning (i.e., I studied > E studied; I studied > I nonstudied (baseline); E studied  =  E nonstudied (baseline)), whereas in the transfer conditions, conscious control over self-generated responses was evident only in the o-m condition, but knowledge was nonetheless above baseline in the m-o condition; and (b) Mean recognition confidence ratings assigned to the 24 six-item sequences revealed accurate recognition memory for the studied sequence in the baseline conditions and o-m transfer condition. Studied sequences were allocated a confidence rating between 1 and 3, whereas nonstudied sequences were allocated a rating between 4 and 6.

Performance on the manual cued-generation task and manual recognition test in o-m transfer condition indicated that oculomotor sequence learning supported effector- and response-independent sequence knowledge. In particular, the participants in the o-m transfer condition were able to exert conscious control over self-generated manual responses on the cued-generation task (i.e., I studied > E (*F*_(1,60)_ = 24.49, *p*<.0001); I studied > I baseline (*F*_(1,60)_ = 15.47, *p*<0.001); E studied  =  E baseline (*F*_(1,60)_ = 2.57, *p* = 0.11), and demonstrated recognition memory for the studied sequence (*t*_(15)_ = 2.33, *p*<0.05), in absence of recognition priming (early (*t*_(15)_ = 0.04, *p* = 0.97); late (*t*_(15)_ = 0.17, *p* = 0.87)) and earlier priming on the inter-modality indirect test (*F*<1) (Figures 3 and 4). By contrast, in the m-o transfer condition, manual sequence learning did not support conscious control over self-generated oculomotor responses on the cued-generation task (I studied  =  E studied (*F*_(1,60)_ = 1.37, *p* = 0.24) or recognition memory for the studied sequence (*t*_(15)_ = −1.10, *p* = .29). Nonetheless, sequence-specific knowledge was evident in the above baseline oculomotor responses in the inclusion and exclusion tests of the cued-generation task (E studied > E baseline (*F*_(1,60)_ = 6.63, *p*<0.05); I studied > I baseline (*F*_(1,60)_ = 6.69, *p*<0.05)) (Figure 4), and in the primed oculomotor responses associated with the “late” predictable positions on the recognition memory test (*t*_(15)_ = 2.48, *p*<0.05; early, *t*_(15)_ = 1.06, *p* = 0.31). The latter data demonstrate that four elements were required to provide sufficient context to induce the priming of oculomotor responses, but this effect did not survive correction for multiple comparisons. Nonetheless, there was evidence of significant priming of oculomotor responses on the inter-modality indirect test (T2), which was administered immediately after the study phase (*F*_(1,30)_ = 16.69, *p*<0.001) (Figure 3).

Notably, there was a significant difference between the manual baseline and manual to oculomotor transfer conditions in the magnitude of general skill (41 ms) acquired on manual SRT task (41 ms, *t*_(30)_ = −2.42, *p*<0.05), but not in the magnitude of sequence-specific knowledge (T1) (*F*_(1,30)_ = 0.10, *p* = 0.75). We examined the consequences of this difference for the transfer of sequence-specific knowledge to post-study tests administered in the same and different modality with respect to initial study. The results from these analyses indicate that the magnitude of general skill acquired on the manual SRT task could not be used to predict performance on the manual or oculomotor post-study tests (see section entitled, General skill acquired on the manual SRT task and relation to sequence-specific manual and oculomotor knowledge). Therefore, general skill acquired on the manual SRT task was not an explanatory variable relevant to understanding the use of knowledge on the post-study tests administered in the same (m-m) or in a different (m-o) modality with respect to initial training.

### Experiment 2 summary: No evidence of transfer to the manual response modality after perceptual learning (p-m condition)

**Figure 5:**
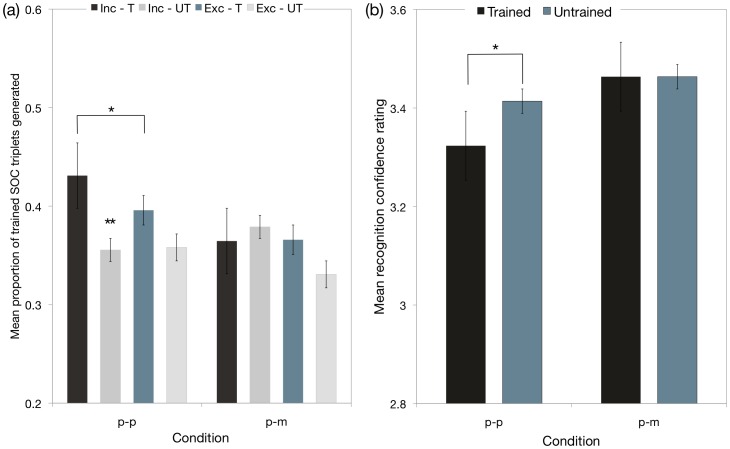
Performance on the cued-generation tasks and recognition tests administered in experiment 2. (a) Mean proportions of studied and nonstudied (baseline) second-order conditional triplets generated for the baseline perceptual cued-generation task and the p-m transfer condition manual cued-generation task, under inclusion and exclusion test instructions; and (b). Mean recognition confidence ratings revealed recognition memory for the studied sequence in the baseline p-p condition (perceptual recognition test), but no evidence of recognition memory in the p-m transfer condition (manual recognition test). Studied sequences were allocated a confidence rating between 1 and 3, whereas nonstudied sequences were allocated a rating between 4 and 6.

**Figure 6:**
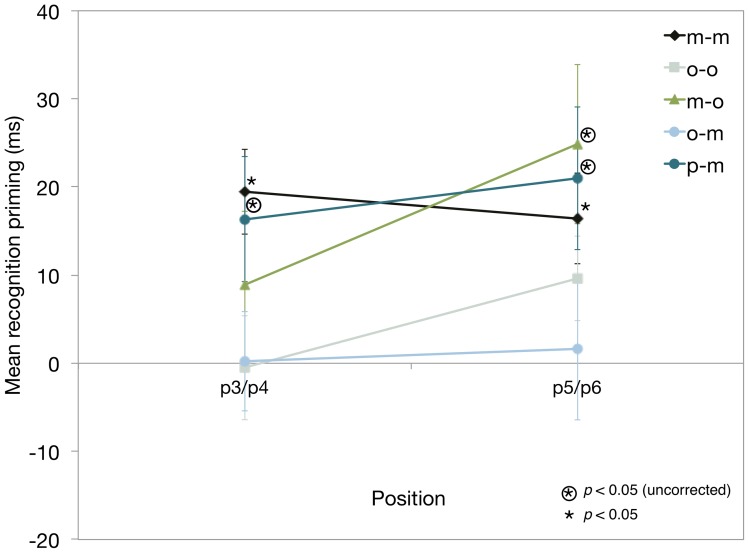
Response priming on the recognition tests that were administered in the baseline and transfer conditions (experiments 1 and 2). The difference in response latencies associated with the six-element studied and nonstudied recognition probes was used to measure priming. Mean response latencies (RTs or saccade latencies) to studied and nonstudied recognition sequences at positions 3–6 (predictable positions) are shown – position 3 represents the earliest point at which the (second-order conditional) response context can induce priming. Mean recognition priming scores provide an indirect (procedural) test of sequence knowledge (latencies associated with responses to studied positions subtracted from the corresponding RTs to nonstudied positions). Results are combined and presented for collapsed “early” 3–4 (p3/p4) and “late” 5–6 (p5/p6) positions.

There was no evidence of significant transfer in the p-m condition. Null effects were obtained on the manual cued-generation task (I studied  =  E studied  =  B (*Fs*<1.1); I studied > E studied (*Fs*<1)) and manual recognition test (*t*(15) = 0.56, *p* = 0.58) (p-m condition) (Figure 5). Furthermore, evidence for primed manual responses on recognition test (early [16 ms], *t*_(15)_ = 2.30, and, late [21 ms], *t*_(15)_ = 2.59, *p's*<0.05) (Figure 6) did not survive correction for multiple comparisons.

### Data reduction: Accuracy, latency, and central fixation

#### Accuracy

The first 4 buffer trials from each block and incorrect manual responses were excluded from analyses performed on the data obtained manual SRT task and manual indirect tests. Correspondingly, the first 4 buffer trials from each block along with mismatch and location error trials were excluded from the analyses performed on data obtained from the oculomotor SRT task and oculomotor indirect tests.

#### Latency

Performance on the manual SRT task was summarised by computing the mean RT in each block for each participant. Correspondingly, saccade latencies on the oculomotor SRT task were summarised by computing the mean saccade latencies in each block for each participant. Saccade latencies were consistently faster than manual RTs, leading to baseline differences between manual and saccade-contingent/oculomotor response modes. Absolute differences in latency are of limited significance because they are a poor measure of sequence knowledge, even when all salient dimensions of study and test conditions are matched [50]. Separate mixed-factorial ANOVAs were used to assess the latency-based measures of sequence-specific knowledge in the oculomotor and manual response conditions [48] (see below).

#### Central Fixation

Analyses of the eye movements recorded during baseline and transfer conditions that involved manual and perceptual responses revealed that participants were able to maintain central fixation. Only a nominal proportion of trials in the manual and perceptual behavioural tasks were associated with saccades (<7%). Notably, since covert shifts of visuospatial attention directed to four locations can be construed as an analogue of a spatially determined oculomotor or a manual response, anticipations of the onset of predictable locations, pre-programmed in advance of the cue to execute a response, are likely to have occurred during study on the perceptual sequence learning task and manual SRT task [24], [51]–[55].

### Experiment 1

#### (1) Study Phase

Figure 3 shows the mean RTs and saccade latencies obtained during m-m and o-o baseline and m-o and o-m transfer conditions. A 2 (response condition: m-m, m-o)×14 (block: 1-14) mixed-factorial ANOVA on the mean RTs revealed a significant main effect of block (*F*_(13,390)_ = 9.19, *p*<0.0001) and a significant interaction between response condition and block (*F*_(13, 390)_ = 2.53, *p*<0.01). The main effect of response condition was not significant (*F*<1).

A 2 (response condition: o-o, o-m)×14 (block: 1-14) mixed-factorial ANOVA on mean saccade latencies obtained from the oculomotor SRT task in the o-o and o-m conditions revealed a significant main effect of block (*F*_(13,390)_ = 3.05, *p*<0.001) and a significant interaction between response condition and block (*F*_(13, 390)_ = 1.77, *p*<0.05), whereas the main effect of response condition was not significant (*F*<1).

#### Manual and oculomotor learning led to significant priming in the baseline and transfer conditions when tested in the same response modality as initial study (T1, Figure 3)

Manual sequence learning in the m-m and m-o conditions led to a significant increase in RT when the non-predictable sequence was presented (T1) (m-m, *F*_(1,30)_ = 16.99, *p*<0.001; m-o, *F*_(1,30)_ = 20.90, *p*<0.001), and the increase in RT did not differ as a function of group (response condition) (*F*_(1,30)_ = 0.10, *p* = 0.75). Correspondingly, significant sequence knowledge was detected after oculomotor sequence learning in the o-o baseline and o-m transfer conditions (o-o, *F*_(1,30)_ = 4.58, *p*<0.05; o-m, *F*_(1,30)_ = 8.39, *p*<0.01), and did not differ as a function of group (*F*_(1,30)_ = 0.29, *p* = 0.59).

#### Manual learning led to significant priming of oculomotor responses, whereas oculomotor learning did not prime manual responses (T2, Figure 3)

In the m-o transfer condition, a 2 (response condition: o-o, m-o)×2 (block: 16-17) mixed-model ANOVA performed on the mean saccade latencies revealed a significant main effect of block (*F*_(1,30)_ = 16.69, *p*<0.001), whereas the main effect of response condition and interaction between response condition and block were not significant (*Fs*<1). Hence, the increase in saccade latency between blocks 16 (studied) and 17 (non-studied/non-predictable sequence) was significant in both the oculomotor baseline condition and m-o transfer conditions, and indicates significant inter-modality transfer of knowledge in the m-o condition.

In the o-m transfer condition, a 2 (response condition: m-m, o-m)×2 (block: 16-17) mixed-model ANOVA performed on the mean RTs revealed a significant interaction between response condition and block (*F*_(1,30)_ = 7.74, *p*<0.01), whereas the main effects of response condition (*F*_(1,30)_ = 2.20, *p* = 0.15) and block (*F*_(1,30)_ = 3.86, *p* = 0.058) were not significant. Planned comparisons conducted to explore the interaction term revealed significant knowledge in the manual baseline condition (*F*(1,30) = 11.27, *p*<0.01), but not in the o-m transfer condition (*F*<1). These results demonstrate that sequence-specific knowledge was detected on the second (manual) indirect test, but there was no significant evidence of manual response priming after oculomotor sequence learning.

#### General skill acquired on the manual SRT task and relation to sequence-specific manual and oculomotor knowledge

The interaction term between response condition and block indicated that there was a significant between-group difference in the magnitude of general skill (41 ms difference) across the 14 blocks on the manual SRT task (i.e., general response execution) (*F*_(13, 390)_ = 2.53, *p*<0.01). However, the difference in general skill did not lead to a significant difference in the magnitude of sequence-specific knowledge acquired in the m-m and m-o conditions, when measured on the first indirect test, T1 (*F*_(1,30)_ = 0.10, *p* = 0.75). Even though the identical study protocol yielded a reliable magnitude of sequence-specific knowledge, we conducted additional exploratory analyses to assess the impact of the variability in general skill on the post-study measures of sequence-specific knowledge within the manual response modality and on the transfer of sequence-specific knowledge to the oculomotor response modality.

First, we examined the time course of general skill acquisition on the manual SRT task by obtaining a composite learning score for epochs involving sequence blocks 2–4, blocks 5–7, blocks 8–10, and blocks 11–13 of the study phase (i.e., the change in general skill related RT associated with the studied sequence, across each epoch). A mixed-model ANOVA performed on the mean composite non-specific learning scores revealed that the main effect of response condition (*F*_(1,30)_ = 1.38, *p* = 0.25) and epoch (*F*_(3,90)_ = 0.82, *p* = 0.48) were not significant, and the interaction between response condition and epoch was also not significant (*F*_(3,90)_ = 0.78, *p* = 0.51). Hence, general skill learning on the manual SRT task was not reliably different between the m-m and m-o groups at each of the four epochs.

Second, to determine whether there was a link between general skill and sequence-specific knowledge on the post-study tests, we calculated a general skill score for the m-m and m-o conditions (i.e., mean RT block 2 minus mean RT block 13). RTs speeded up more in the m-m baseline condition (55 ms, S.E.M.  = 10.9) as compared to m-o transfer condition (14 ms, S.E.M.  = 13.2) (Figure 3), and the 41 ms difference was significant (*t*_(30)_ = −2.42, *p*<0.05). We then determined whether the magnitude of general skill could be used to predict the magnitude of sequence-specific knowledge on the manual post-study tests. In the m-m condition, the magnitude of general skill did not predict the magnitude of sequence-specific knowledge on the indirect tests (T1 (β = 0.14, *t* = 0.53, *p* = 0.60; T2 (β = −0.12, *t* = −0.45, *p* = 0.66)), the magnitude of above baseline knowledge on the inclusion (β = −0.27, *t* = −1.07, *p* = 0.30) and exclusion (β = −0.12, *t* = −0.46, *p* = 0.66) tests of the cued-generation task, recognition memory for the studied sequence (β = 0.27, *t* = 1.06, *p* = 0.31), or the magnitude of response priming on the manual recognition test for early (β = 0.44, *t* = 1.83, *p* = 0.09) and late (β = 0.29, *t* = 1.14, *p* = 0.27) positions. Similarly, in the m-o condition, general skill did not predict the magnitude of sequence-specific knowledge on the intra-modality indirect test (T1) (β = 0.11, *t* = 0.41, *p* = 0.68). These data demonstrate that the magnitude of general skill acquired on the manual SRT task was not associated with the magnitude of sequence-specific knowledge measured on the manual post-study tests.

In the m-o condition, we examined whether there was a link between general skill acquired on the manual SRT task and sequence-specific knowledge measured on oculomotor post-study tests. The t-statistics were not significant at the 0.05 critical alpha level for the inter-modality indirect test (T2) (β = −0.45, *t* = −1.88, *p* = 0.08), the magnitude of above baseline performance on the inclusion (β = −0.19, *t* = −0.72, *p* = 0.48) and exclusion (β = −0.10, *t* = −0.39, *p* = 0.70) tests, and recognition memory for the studied sequence (β = 0.05, *t* = 0.19, *p* = 0.85). Interestingly, the magnitude of general skill predicted the sequence-specific oculomotor priming of RTs for early (β = −0.64, *t* = 3.10, *p* = 0.008) and late (β = −0.70, *t* = −3.64, *p* = 0.003) positions on the recognition test.

In summary, the results indicate that the between-group difference in general skill was not reliable during the study phase (as assessed by the epoch analysis), and was only significant across the entire study phase, which involved three types of block (pseudorandom, target sequence blocks and a non-predictable block). Furthermore, there was no evidence for a relation between general skill and the multiple post-study dependent measures administered in the manual response modality. With the exception of response priming on the oculomotor recognition test, there was also no significant relation between general skill and sequence-specific knowledge on the oculomotor post-study tests. It is nonetheless conceivable that larger general skill scores – as found in the m-m condition – may be relevant to explaining performance on the oculomotor post-study tests. However, this is unlikely for several reasons. First, we failed to find a relation between the magnitude of general skill and sequence-specific knowledge on the intra-response modality post-study tests; i.e., under conditions where the overlap of task-relevant operations and information between study and test were greater than those associated with transfer from the manual SRT task onto the oculomotor post-study tests. Therefore, given the substantial informational and task differences between general skill learning on the manual SRT task and the expression of sequence-specific knowledge in the context oculomotor recognition priming, the link may be more apparent than real. Second, the only significant coefficient in the m-o condition was between general skill and oculomotor recognition priming, whereby larger general skill scores were associated with less response priming on the oculomotor recognition test. Importantly, this link involved a priming effect that was not significant for corrected p-values, and, there was no evidence for a significant relationship between general skill and the (significant) sequence-specific priming detected on the second (oculomotor) indirect test, T2. Together, the additional analyses objectively demonstrate that an equivalent magnitude of general skill on the manual SRT task pre-transfer was not required to study transfer in the m-o condition, because the candidate variable – general skill – had no effect on the dependent measures that operationalised our construct of interest, via RT, identification, and production based measures.

#### (2) Direct tests: (a) Awareness of the sequence measured on the post-study questionnaire was equivalent across all response conditions

Mean sequence awareness questionnaire ratings were calculated for each response condition: o-o (M = 3.0, SE  = .28), m-m (M = 2.1, SE  = .28), o-m (M = 2.75, SE  = .28), and m-o (M = 2.4, SE  = .28). A one-way ANOVA revealed no significant effect of response mode (*F*_(3,60)_ = 2.16, *p* = 0.10). Hence, the level of subjective awareness was equivalent across the four response conditions, despite, for example, the difference in general skill between the m-m and m-o conditions, and, more broadly, the fundamental differences in informational and task demands associated with each response modality.

#### (2) Direct tests: (b) Cued-generation tasks (Figure 4a)

Performance was assessed by first calculating the proportion of SOC sequence triplets generated out of the maximum number of correct SOC triplets that could be generated [28]. Four possible triplets could be generated on each of the 12 trials per test, thereby yielding a possible 48 correct SOC triplets on each test. Baseline rates of responding were calculated separately for each test as the proportion of triplets that were inconsistent with the studied sequence; i.e., the proportion of triplets from the nonstudied sequence.

A 4 (response condition: m-m, o-o, m-o, o-m)×2 (test: inclusion, exclusion)×2 (sequence: studied, nonstudied [baseline]) mixed-factorial ANOVA performed on the mean proportions of triplets generated on the cued-generation task revealed a significant main effect of test (*F*_(1,60)_ = 22.41, *p*<0.001), sequence (*F*_(1,60)_ = 26.12, *p*<0.001), and significant interactions between test and sequence (*F*_(1,60)_ = 29.91, *p*<0.001), and response condition, test and sequence (*F*_(3,60)_ = 2.97, *p*<0.05). No other main effects or interactions were significant (*Fs*<1.4).

Above baseline knowledge was detected in both transfer conditions, but conscious control was evident only in the o-m transfer condition. Planned comparisons revealed that manual sequence learning (m-o transfer condition) supported above-baseline self-generated oculomotor responses on the cued-generation task (E studied > E baseline (*F*_(1,60)_ = 6.63, *p*<0.05); I studied > I baseline (*F*_(1,60)_ = 6.69, *p*<0.05), but failed to support conscious control (I studied  =  E studied (*F*_(1,60)_ = 1.37, *p* = 0.24)). By comparison, planned comparisons revealed that oculomotor sequence learning led to effector- and -response-independent knowledge that could be deployed on the manual cued-generation task: participants were able to exert conscious control over self-generated manual responses (i.e., I studied > E studied (*F*_(1,60)_ = 24.49, *p*<0.0001); I studied > I baseline (*F*_(1,60)_ = 15.47, *p*<0.001); E studied  =  E baseline (*F*_(1,60)_ = 2.57, *p* = 0.11)).

In the m-m and o-o baseline conditions, participants were able to exert conscious control over self-generated responses guided by the studied sequence. In particular, planned comparisons revealed that manual sequence learning supported consciously controlled responses on the manual cued-generation task (i.e., I studied > E studied (*F*_(1,60)_ = 13.30, *p*<0.001); I studied > I baseline (*F*_(1,60)_ = 14.91, *p*<0.001); E studied  =  E baseline (*F*<1)). Similarly, oculomotor sequence learning supported conscious control over responses determined by the studied sequence on the oculomotor cued-generation task (i.e., I studied > E studied (*F*_(1,60)_ = 12.06, *p*<0.001); I studied > baseline (*F*_(1,60)_ = 12.74, *p*<0.001); E studied  =  E baseline (*F*_(1,60)_ = 1.71, *p*>0.05)).

#### Cued-generation task awareness questionnaire

A 4 (response condition: m-m, o-o, m-o, o-m)×2 (test: inclusion, exclusion) mixed-factorial ANOVA performed on the mean subjective ratings of performance on the inclusion and exclusion tests revealed a significant main effect of test (*F*_(1,60)_ = 8.29, *p*<0.01), whereas the main effect of response mode and interaction between response mode and test were not significant (*Fs*<1.1). These results indicate that across all conditions the confidence ratings on the inclusion test (M = 32, SE  = 3.22) were consistently lower than those associated with the exclusion test (M = 41, SE  = 3.38).

#### (2) Direct tests: (c) Recognition memory tests (Figures 4b and 6): Accuracy and Priming

Accuracy: Recognition memory for the studied sequence was detected in the o-m but not in the m-o transfer condition (Figure 4b). In particular, analysis of the mean recognition confidence ratings in the o-m transfer condition revealed that oculomotor sequence learning supported accurate memory for the studied sequence on the manual recognition test (*t*_(15)_ = 2.33, *p*<0.05). By contrast, in the m-o transfer condition, participants were unable to discriminate between studied and nonstudied sequences on the oculomotor recognition test (m-o transfer condition), (*t*_(15)_ = −1.10, *p* = .29). The differences in mean confidence ratings assigned to old and new sequences in the oculomotor and manual baseline conditions were consistent with accurate recognition memory for the studied sequence (*t*_(15)_ = −4.64, *p*<0.001 and *t*_(15)_ = 3.98, *p*<0.001, respectively).

Priming: Significant priming was obtained only in manual baseline condition (Figure 6). In particular, manual RTs and saccade latencies to each six-element studied and nonstudied sequence provided an indirect measure of response priming related to the studied sequence [35], [56], and the analysis was predicated on the notion that if learning had taken place, responses to predictable (studied) stimuli (positions 3–6) would be faster than the latencies associated with unpredictable (nonstudied) stimuli [35] –only positions 3–6 were predictable from the preceding targets. Figure 6 shows the results of the priming score calculated by subtracting the mean latencies for studied sequences from nonstudied sequences for positions 3–6; these values were averaged for each position (3–6) and then across (early) positions 3 and 4 and across (late) positions 5 and 6 [36]. Early and late positions were analysed to determine the amount of context needed to facilitate response priming [28], [36]. Repeated measures t-tests were performed against chance (0) to determine whether or not there was significant recognition priming in the baseline and transfer conditions (Figure 6).

Results from the o-m transfer condition indicated that oculomotor sequence learning did not support the priming of manual responses to the studied sequence (early (*t*_(15)_ = 0.04, *p* = 0.97); late (*t*_(15)_ = 0.17, *p* = 0.87)), whereas in the m-o transfer condition, manual sequence learning supported the priming of oculomotor responses to the studied sequence in the late predictable positions (*t*_(15)_ = 2.48, *p*<0.05; early, *t*_(15)_ = 1.06, *p* = 0.31); however, the priming effect was not significant for the Bonferroni-Holm corrected p-values [57].

In the m-m baseline condition, significant priming was obtained in the “early” locations 3–4 (i.e., 3–4 (*t*_(15)_ = 4.02, *p*<0.01)) and “late” locations 5–6 (*t*_(15)_ = 3.21, *p*<0.01), whereas in the o-o baseline condition, there was no evidence of significant priming in the mean saccade latencies to the studied sequence (early (*t*_(15)_ = −0.08, *p* = 0.94); late (*t*_(15)_ = 2.02, *p* = 0.06)). T-tests were significant for Bonferroni-Holm corrected p-values [57].

### Experiment 2

#### (1) Study phase: performance on LDT counting task was at ceiling

Performance on LDT counting task was consistent with a level of automaticity that would have allowed resources to be directed at perceptual learning [58], because the error rate was less than 5% (mean error  = 4.6%). Importantly, behavioural evidence indicates that secondary tasks such as tone counting disrupt performance, but not learning, on the manual SRT task [59]; that is, learning on the manual SRT task is often minimally affected by cognitive load [59], and previous work indicates that there is no interaction between the error rate on the LDT counting task and manual or perceptual sequence learning [28]. From a neurocognitive perspective, learning two sequences simultaneously does not appear to alter the neural activity in a substantive manner [60]. Concurrent tasks are argued, however, to the limit the availability of conscious knowledge [50], [61], but, in the perceptual baseline condition, the LDT counting task did not preclude reliable above baseline knowledge and the use conscious control on the cued-generation task, nor did it preclude recognition of the studied sequence (see below).

#### (2) Direct tests: (a) Perceptual learning was not associated with subjective awareness of the sequence

In the p-m transfer condition, the mean questionnaire rating was 1.7 (S.E.  = 0.29), whereas in the p-p condition the mean rating was 1.2 (S.E.  = 0.10). The difference was not significant (*t*_(30)_ = 1.59, *p* = 0.12), and is in line with the use of an identical study protocol across both conditions.

#### (2) Direct tests: (b) Cued-generation task (Figure 5a)

The mean proportions of triplets generated in the baseline condition and perceptual to manual transfer condition are shown in Figure 5a. A 2 (response condition: p-p, p-m)×2 (test: inclusion, exclusion)×2 (sequence: studied, baseline) mixed-factorial ANOVA performed on the mean proportion of generated triplets revealed a significant main effect of sequence (*F*_(1,30)_ = 5.11, *p*<0.05), whereas the main effects of response condition and test were not significant (*F*_(1,30)_ = 3.53, *p* = 0.07, and, *F*_(1,30)_ = 3.27, *p* = 0.08, respectively). Importantly, there was a significant interaction between response condition, test, and sequence (*F*_(1,30)_ = 4.33, *p*<0.05). No other interactions were significant (*Fs*<1.4).

Above baseline knowledge and conscious control were found only in the p-p baseline condition. In particular, planned comparisons revealed that perceptual sequence learning did not lead to above-baseline knowledge on the manual cued-generation task (I studied  =  E studied  =  B (*Fs*<1.1); I studied > E studied (*Fs*<1)), whereas in the baseline condition, perceptual learning supported conscious control over self-generated responses (I studied > E studied (*F*_(1,30)_ = 6.71 *p*<0.01)); I studied > I baseline (*F*_(1,30)_ = 10.47, *p*<0.01); E studied  =  E baseline (*F*<1.0)).

#### Cued-generation task awareness questionnaire

A 2 (response condition: p-p, p-m)×2 (test: inclusion, exclusion) mixed-model ANOVA performed on the mean subjective ratings of performance on the inclusion and exclusion tests revealed a significant main effect of test (*F*_(1,30)_ = 4.33, *p*<0.05), whereas the main effect of response condition and interaction between response condition and test were not significant (*Fs*<1). These results indicate that the confidence associated with performance on the inclusion test (M = 29, SE  = 3.65) was consistently lower than that assigned to the exclusion test (M = 37, SE  = 4.38). Interestingly, therefore, learning on the perceptual sequence learning task led to little or no meta-knowledge about the studied sequence, despite the evidence of conscious controlled sequence-specific knowledge on the perceptual cued-generation task.

#### (2) Direct tests: (c) Recognition memory for the studied sequence was confined to the p-p baseline condition (Figure 5b)

In the perceptual baseline condition, sequence-specific knowledge was evident in the ability to recognise the studied sequence (*t*_(15)_ = 2.32, *p*<0.05) (see Figure 4b). By contrast, the confidence ratings associated with old and new sequences did not reveal evidence of accurate recognition memory in the perceptual to manual transfer condition (*t*_(15)_ = 0.56, *p* = 0.58). Furthermore, although the RTs associated with early (16 ms) and late (21 ms) positions were suggestive of response priming on the recognition test (*t*_(15)_ = 2.30 and late, *t*_(15)_ = 2.59, *p's*<0.05, respectively), these effects were not significant for Bonferroni-Holm corrected p-values [57].

## Discussion

The aim of the current study was to examine the ability to generalise new knowledge acquired after learning a visuospatial sequence via responses based on either manual key presses, obligatory eye movements, or covert reorienting of visuospatial attention. The ability to generalise new sequence knowledge was assessed by administering post-study direct and indirect tests either in the same (baseline conditions) or a novel response modality (transfer conditions) with respect to initial study. We focus on the two main results that advance our understanding of the flexibility underlying new sequence knowledge. First, learning in the baseline conditions led to the conscious use of sequence-specific knowledge on the cued-generation task and accurate recognition memory. Hence, the ability to use the products of learning flexibly on these direct tests was comparable across manual, oculomotor, and perceptual response modalities. Second, the results from the transfer conditions indicated that the ability to generalise new knowledge was affected by the responses used to initially code the visuospatial sequence. Oculomotor sequence learning was associated with conscious control over self-generated manual responses on the cued-generation task and accurate recognition memory, but did not support the priming of manual responses. By contrast, manual sequence learning only supported above baseline knowledge on the oculomotor cued-generation task; however, these self-generated oculomotor responses were not subject to conscious control.

Transfer onto the manual post-study tests after perceptual sequence learning was detected only in the priming of manual responses on the recognition test, but this did not survive correction for multiple comparisons. Therefore, even though perceptual sequence learning supported the conscious use of new sequence knowledge on the cued-generation task and accurate recognition memory, there was no evidence to indicate that perceptual learning could be applied to guide sequence-specific responding in the manual modality. Hence, the informational content associated sequence knowledge acquired via perceptual learning was orthogonal to the distinction between conscious and nonconscious knowledge [62]. By inference, the state of awareness associated with sequence knowledge does not appear to predict whether new sequence knowledge can be deployed outside of the original effector and responses associated with learning; the data are, thereby, at variance with neurobiological accounts of sequence learning that have been derived from experimental studies in the manual modality [63].

Notably, prior to transfer, the manual baseline and the manual to oculomotor transfer conditions differed in terms of the magnitude of general skill acquired across the study phase. Importantly, however, the magnitude of general skill did not predict the magnitude of (significant) sequence-specific knowledge measured on the post-study tests in either condition. Furthermore, the rate general skill learning on the manual SRT task was not reliably different between the manual baseline and manual to oculomotor transfer condition when it was examined at the level of four epochs. Therefore, the variability in general skill was not relevant to understanding the results obtained on the post-study tests of sequence-specific knowledge in either of these conditions.

### Representational flexibility of new sequence knowledge on tests administered in the same response modality as initial study

In the manual and perceptual baseline conditions, the ability to exert conscious control over self-generated responses along with accurate recognition memory for the studied sequence replicated the results that we reported in a previous study [28]. In line with this level of representational flexibility, oculomotor sequence learning supported accurate recognition memory and conscious control over self-generated responses on the oculomotor cued-generation task. These data stand in contrast to previous studies of oculomotor sequence learning that have failed to find evidence of robust sequence-specific knowledge on direct tests [6], [29]. Even though the informational demands on many of our dependent measures did not overlap with the oculomotor SRT task, sensitivity to the available sequence knowledge may have been enhanced by the use of direct tests that were also dependent responding via obligatory saccade-contingent responses. More broadly, the evidence of significant sequence-specific knowledge on the manual, oculomotor, and perceptual versions of the post-study tests demonstrates that the performance obtained in the transfer conditions is unlikely to reflect a loss of information or response-based differences in the sensitivity of these tests to the available knowledge [19]. For example, the absence of conscious control over self-generated oculomotor responses on the cued-generation task after manual sequence learning (i.e., in the m-o condition) cannot be attributed to a general failure to exert conscious control when responding with eye movements (cf. o-o baseline condition).

Several broader issues pertain to nature of sequence knowledge acquired outside of the manual modality. First, numerous studies have reported that the response-to-stimulus interval can affect properties such as knowledge about local statistical structure or the state of awareness associated with the new sequence knowledge [64]–[67]. Here, the manual and oculomotor baseline conditions were distinguished by a small difference in the mean inter-stimulus interval. Nonetheless, the difference in the ISI, alongside the fixed 1200 ms response-to-stimulus, did not have a measurable impact on the performance in the direct tests, inasmuch as sequence-specific knowledge was detected on the cued-generation tasks and recognition tests after manual and oculomotor sequence learning. Indeed, response-to-stimulus intervals of longer than 250 ms do not appear to have a measurable difference on the availability of sequence-specific knowledge, at least on the manual SRT task [68]. Second, systematic investigation using temporal parameters such as the response-to-stimulus interval will be required in order to pinpoint the specific role of visuospatial anticipatory mechanisms, working memory, and conscious access in determining the generalisability of new sequence knowledge, when acquired by oculomotor and perceptual sequence learning [37], [68].

### Generalisation of new sequence knowledge to a novel response modality

Sequence knowledge acquired in the manual to oculomotor and oculomotor to manual transfer conditions was detected on at least one of the post-study indirect or direct tests. Transfer to these tests did not depend on the reinstatement of a learned motor program, because the use of knowledge always involved kinematic and spatial transformations between study and test. Evidence of significant transfer after oculomotor sequence learning is consistent with the coding of stimulus locations [62], [69], [70], and implies global access to the knowledge [71]. By contrast, the absence of conscious control over manual responses on the cued-generation task and failure to recognise the studied sequence after manual sequence learning is suggestive of reduced representational flexibility, due to the coding of low-level motor commands, coupled to specific effectors or response-specific information [72], [73]. Both the response-effect distinctiveness, related to proprioceptive and auditory feedback, and the lower stimulus-response compatibility associated with the manual responses may have led to more rapid sequence learning [74], [75], and thereby the emergence of effector and response-specific knowledge through extended rehearsal [16], [41], [42], [76]. It may be possible to restrict effector-specific and response-specific knowledge using a training protocol based on a probabilistic visuospatial sequence, because the point at which sequence-specific knowledge emerges (probable RTs < improbable RTs) can be determined and then used to limit over extended rehearsal [28], [77], [78]. Notably, however, manual sequence learning was not entirely effector-specific or response-specific, because performance on the inclusion and exclusion tests was above baseline.

The failure to detect sequence knowledge on the manual indirect and direct tests after perceptual learning is consistent with the coding of response-specific information. In particular, although manual responses on the recognition test were associated with priming, with only a minimal amount of contextual support, the effect did not survive correction for multiple comparisons. Hence, performing motor responses to the target visuospatial sequence may be a necessary feature for the use of sequence-specific knowledge on visuomotor direct tests [72], [79]–[81]. Key components of knowledge that are missing from perceptual sequence learning, and which are arguably relevant to successful transfer, include integrated motor action-effect and motor plan-based information, optimised motor attention and spatial response selection, and experience with the synchronization of motor responses to the visual stimuli [1], [82]–[87]. Future studies could assess the apparent necessity of motor responses by determining if transfer after perceptual sequence learning is similarly limited when the target sequence is based on first-order associations, because such simple pairwise associations are qualitatively distinct from the higher-order visuospatial sequences that were studied here [7].

## Conclusions

The results demonstrated that the responses used to code for a higher-order visuospatial sequence affected the ability to generalise new sequence knowledge in a novel response modality, but did not have a conspicuous impact on the use of knowledge on tests that were administered in the same response modality as initial study. The evidence of asymmetric transfer between manual and oculomotor modalities is suggestive of distinct neuronal processes supporting each form of learning [2]. At present, however, functional neuroimaging based investigation of the mechanisms responsible for representational flexibility and specificity have been confined to the manual effector and the transfer of first-order associations [9], [12], [13], and will need to be elaborated by modeling based approaches [88]. Hence, the network of brain regions and basic mechanisms that support the representational flexibility necessary to generalise higher-order visuospatial sequence knowledge across different response modalities are not well understood and remain to be established.
